# Effect of Probiotic Streptococcus salivarius K12 and M18 Lozenges on the Cariogram Parameters of Patients With High Caries Risk: A Randomised Control Trial

**DOI:** 10.7759/cureus.23282

**Published:** 2022-03-18

**Authors:** Saravanan Poorni, MS Nivedhitha, Manali Srinivasan, Arthi Balasubramaniam

**Affiliations:** 1 Conservative Dentistry and Endodontics, Saveetha Dental College and Hospital, Chennai, IND; 2 Conservative Dentistry and Endodontics, Sri Venkateswara Dental College, Chennai, IND; 3 Community Dentistry, Saveetha Dental College and Hospital, Chennai, IND

**Keywords:** lozenge, probiotics, streptococcus salivarius, caries risk, cariogram

## Abstract

Purpose: To evaluate the effect of Probiotic *Streptococcus Salivarius* K12 and M18 Lozenges on the Cariogram (Cariogram Mobile application Version 1.3 developed by Wong Jung Ming, Faculty of Dentistry, National University of Singapore) parameters of patients with high caries risk.

Materials and Methods: Fourty-two subjects were randomly allocated to Group 1 and 2 who received BLIS K12^TM ^and BLIS M18^TM ^probiotics (Blis Probiotics, Dunedin, New Zealand) respectively along with oral hygiene instructions and Group 3 (control) received only oral hygiene instructions with 1:1:1 allocation ratio. Subjects were instructed to follow the instructions and use the probiotics for a period of three months. Their caries risk was assessed using Cariogram software at baseline and 30 days after the use of probiotics. Change in the chance to avoid new cavities was recorded and statistically analysed using appropriate statistical tests.

Results: About 38 subjects completed the trial with a drop-out count of eight. Multiple imputations were carried out for the missing data using an expectation-maximization algorithm. The mean percentage of actual chance to avoid new cavities was found to be 47.14 ± 6.837; 41.36 ± 16.04 and 32.50 ± 14.54 among the subjects in Group 1, Group 2, and Group 3 respectively. The mean percentage difference between the three groups was found to be statistically significant (p-value = 0.047). Dunn’s pair-wise comparison showed a significant mean percentage difference between Group 1 (BLIS K12) and Group 3 (control) (p=0.020).

Conclusion: It can be concluded that the use of BLIS K12^TM ^and BLIS M18^TM ^probiotics for three months resulted in a considerable decrease in the caries risk. Further long-term clinical trials are needed to evaluate the difference in caries risk following the use of BLIS K12^TM^and BLIS M18^TM^ among different age and risk groups.

## Introduction

Dental caries, one of the most prevalent diseases around the world, is characterized by decalcification of the inorganic portion and destruction of the organic matrix of the tooth. Three major factors that contribute towards the progress of dental caries are host, fermentable carbohydrates, and acid-producing bacteria [1,2]. Methods that focus on regulating these factors will help to control the disease's progress.

In recent years, caries risk assessment has gained a lot of attention as a principal method in dental caries management. Caries Risk Assessment is a process that is used to establish the probability of a patient developing a new carious lesion [3,4]. Cariogram is an algorithm-based software that is based on various risk factors such as diet, saliva, oral hygiene, fluoride exposure, past carious experience, and general health of the patient [4,5]. This program demonstrates a multifactorial background of dental caries by illustrating the interaction of nine different caries-related factors enlisted earlier [6].

*Streptococcus mutans* is the most important cariogenic microorganism that is involved in the initiation of the disease. Various methods have been employed to reduce the activity of this bacteria. One such method is the use of probiotics which according to the World Health Organisation (WHO) and the US Food and Drug Administration (FDA) when consumed in adequate levels confers health benefits to the host [7]. The mechanism of action of probiotics is that it acts by inhibiting the multiplication of diverse regular resident microorganisms [8].

Though there are innumerable studies in the literature that have proved the efficacy of probiotics on salivary *Streptococcus mutans* counts [9-11]. However, very few studies have concentrated on the effects of various probiotics on the Cariogram parameters of high caries risk patients. Thus, the current research was undertaken to evaluate the effect of Probiotic *Streptococcus salivarius* K12 and M18 Lozenges on the Cariogram parameters of patients with high caries risk.

## Materials and methods

Study design

After obtaining the approval from the Institutional Ethics Committee of Sri Venkateswara Dental College and Hospital (approval number SVDC/IRB/8E/2019), this randomised double-blinded clinical trial was undertaken to evaluate the changes in the Cariogram parameters following the use of probiotic *Streptococcus salivarius* K12 and M18 Lozenges. The clinical trial was conducted in accordance with the 1964 Declaration of Helsinki and adhered to the CONSORT statement. The trial was undertaken in the Department of Conservative Dentistry and Endodontics, Sri Venkateswara Dental College and Hospital, Chennai for a period of 90 days starting from December 2019. Written informed consents were obtained from all the participants who voluntarily enrolled for the trial.

Study subjects

Prior to the commencement of this clinical trial, 178 subjects were examined and assessed for eligibility. Subjects belonging to the age group of 18-40 years only were examined for eligibility. Subjects with a Decayed Missing and Filled Teeth (DMFT) score of 4 and above were only included for the clinical trial. Subjects with systemic problems, periodontal diseases, allergic conditions, patients who were on antibiotic therapy, and those consuming probiotics for other systemic conditions were excluded from the study. Subjects were also asked to refrain from consuming other probiotics during the trial period.

Sample size

The pilot study was conducted and sample size calculation was done based on the results of the pilot study. Assuming the significance of 5% and 90% chance of detecting the effect size, a sample size of 30 subjects was required. An attrition rate of 20% was expected as it was 90 days trial. Hence 42 subjects were enrolled for the study.

Study proforma

A proforma was constructed to record the patient details. The first part of the proforma consisted of the demographic details of the subjects while the second part consisted of details of 10 Cariogram parameters: Caries Experience, General Disease, Diet Content, Diet frequency, Plaque amount, Mutans Streptococci count, Fluoride therapy, Saliva secretion, Salivary Buffer Capacity, and Clinical Judgement. Every criterion was given scores based on the Cariogram manual [12].

Clinical examination

A thorough clinical examination was done by a single examiner to assess the caries experience of the subject using Decayed, Missing, Filled Surface index (DMFS) as described by WHO 1989. Plaque amount was assessed using Modified Plaque Index as described by Loe H in 1967 [5].

Estimating the salivary parameters

Estimation of the salivary flow rate was done by collecting unstimulated saliva samples. Salivary sample collection was done between 10 am to 12 pm for all subjects. They were asked to spit into a graduated sterile sample collection container every 1 minute for 5 minutes. The salivary flow was recorded in ml/min after 5 minutes. Salivary Buffer Capacity was evaluated using the titration method. Calibration was done using standard pH pellets of pH 4.0 and 7.0. Test samples were then subjected to titration using 250 μL of lactic acid and the pH values of the titrated sample were noted.

Microbial analysis

Mitis Salivarius agar (selective agar for *Streptococcus mutans*) (HiMedia, Mumbai, India) and Rogosa SL agar (Lactobacilli medium) (HiMedia, Mumbai, India) were used for the microbial analysis in the current trial. An inoculation loop was used to streak the sample on the agar medium. The Petri dishes were incubated at 37 degrees Celsius for 48 hours. The organisms were identified based on the morphology, and the Colony Forming Units (CFU) were counted manually. Colony counts were expressed in the number of CFU per ml of saliva.

Constructing caries risk profile using Cariogram

A caries risk profile was obtained for every subject using Cariogram mobile application version 1.3. For each subject, all 10 caries-related parameters were entered. Each parameter was scored based on the key given in the Cariogram manual [12]. For all subjects, the clinical judgement factor was scored as 1. Based on these scores, the chance of avoiding caries was calculated.

Before intervention

During the commencement of the trial, carious teeth were restored for all the subjects enrolled for the trial. Subjects were instructed to follow their routine oral hygiene maintenance and dietary pattern. The baseline data on all 10 Cariogram parameters were recorded and Cariograms were constructed. Following this, the percentage of each of the five sectors of the Cariogram was recorded individually.

Randomisation

Fourty-two subjects enrolled for the study were randomly divided into three groups of 14 subjects each. Random allocation was done using random allocation software, version 2.0 (developed by Department of Anesthesia, Isfahan University of Medical Sciences, Isfahan, Iran). The randomisation ratio was set at 1:1:1. Allocation concealment was done using a sealed envelope to reduce selection bias. Random allocation and allocation concealment were done by a single investigator and were not related to the investigator who examined the subjects and assessed their Cariogram parameters.

Blinding

The study participants in both groups were blinded using an analogous lozenge container. Also, the single calibrated examiner was also blinded and examined for all the 10 Cariogram parameters. 

Intervention

BLIS K12^TM^ and Throat Health BLIS M18^TM^ which were the two forms of commercially *Streptococcus salivarius* were used for the trial. Forty-two subjects were randomly allocated to three groups of 14 subjects each. Subjects belonging to Group 1 received BLIS K12^TM^, while subjects belonging to Group 2 received BLIS M18^TM^, and subjects belonging to Group 3 did not receive any form of probiotics. Subjects in Groups 1 and 2 were instructed to take one lozenge of probiotics for 90 consecutive days. They were instructed to hold the probiotic under the tongue during bedtime, especially after brushing their teeth. Post ingestion of probiotics, the subjects were instructed not to eat or drink for 30 minutes. Subjects were recalled every 30 days and a fresh set of probiotics was issued once in 30 days, and they were also asked to return the unused tablets, if any, at the end of 30 days. This was done to evaluate their adherence level and a 95% adherence rate was considered acceptable. Subjects belonging to the control group did not receive any treatment and were asked to follow their regular oral hygiene procedures and dietary patterns. At the end of the 90 days trial, the Cariogram parameters were recorded for all the subjects in all three groups. The 10 Cariogram parameters were entered in the application and the Cariogram was constructed post-intervention. The data of each of the five sectors were again recorded post-intervention for all subjects.

Statistical analysis

Data obtained at baseline and following 90 days' use of probiotics was recorded in Microsoft Office Excel 2016. The change in the green sector of the Cariogram that corresponded to the percentage of chance to avoid new caries was then calculated for the three groups. The data was then subjected to statistical analysis using SPSS software version 23.0 (IBM Corp., Armonk, NY). Numerical normality test such as the Shapiro-Wilk test was used to explore normal distribution. The data were found to be not normally distributed. Hence, non-parametric tests such as the Kruskal-Wallis test and Wilcoxson signed-rank test were done to compare percentage chance to avoid new cavities between and within groups respectively. Dunn’s pair-wise comparison between groups was carried out. A p-value <0.05 was considered to be statistically significant.

## Results

**Figure 1:**
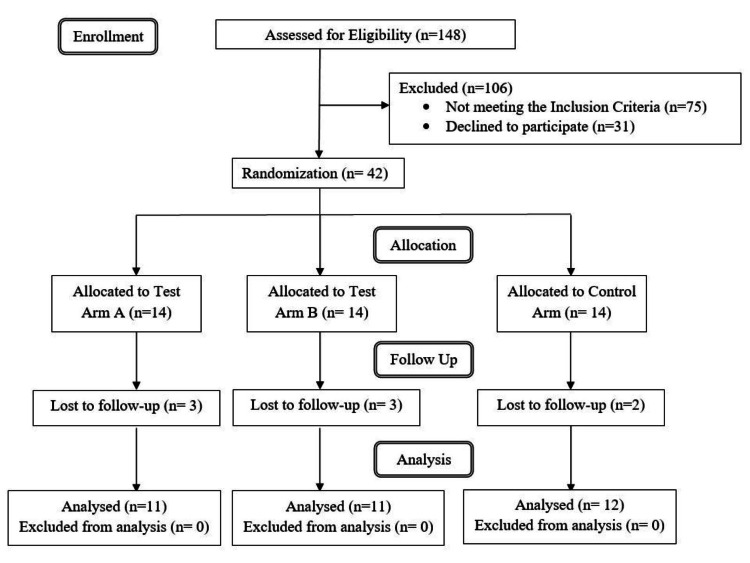
Flow diagram for recruitment of participants

This randomised control clinical study was designed to evaluate the effect of probiotic *Streptococcus salivarius* K12 and M18 Lozenges on the Cariogram parameters of patients with high caries risk. The study was conducted on 42 subjects who were at a high risk of developing new caries. Among the 42 subjects enrolled, 34 subjects completed the trial, eight subjects were lost to follow up in the three months. Multiple imputation was carried out for the missing data using the expectation-maximization algorithm to avoid bias in reporting results. Figure 1 shows the details on the number of subjects enrolled for the trial, the number of subjects who completed the trial in each group, and the information on subjects who were excluded from the trial. The mean age of the participants across all groups was 26.77 ± 5.37. A total of 26 males and 15 females participated in the study.

**Figure 2:**
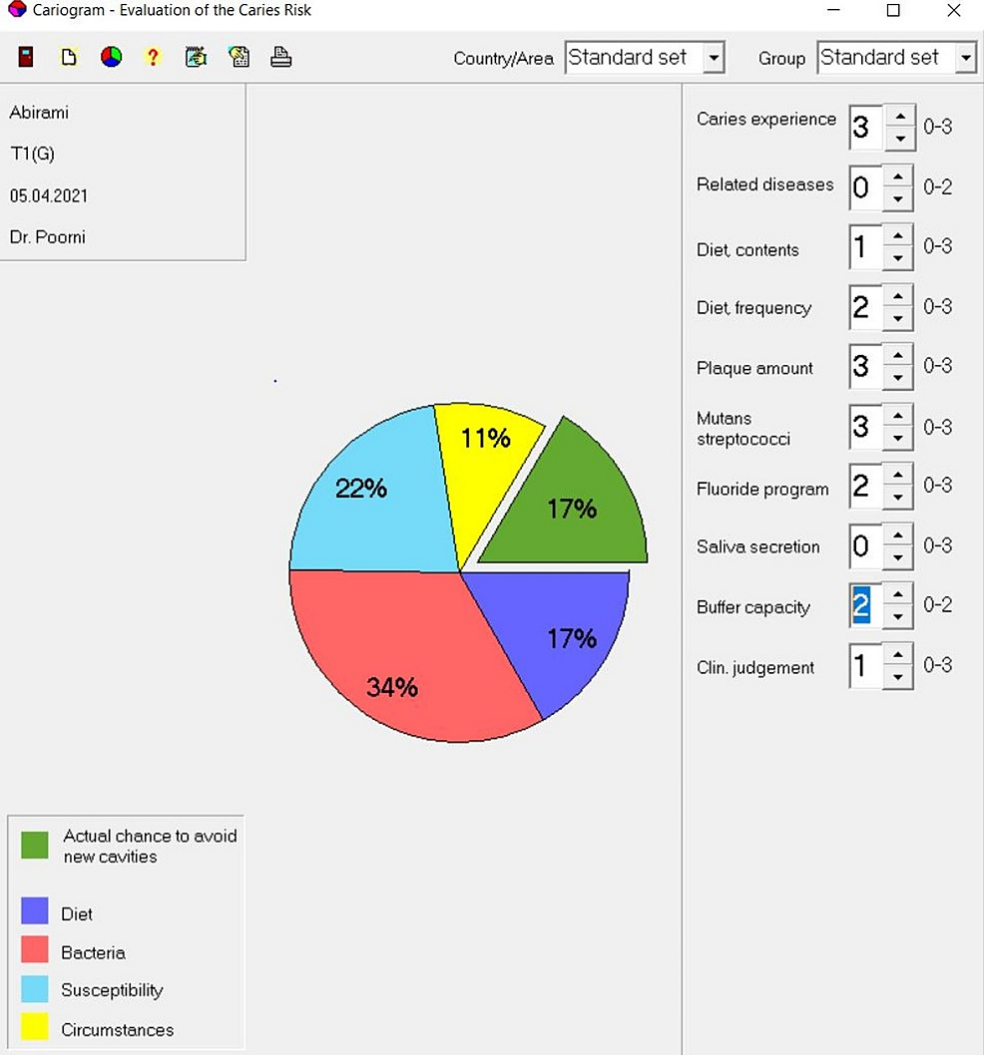
Baseline Cariogram Sector (Group 1)

**Figure 3:**
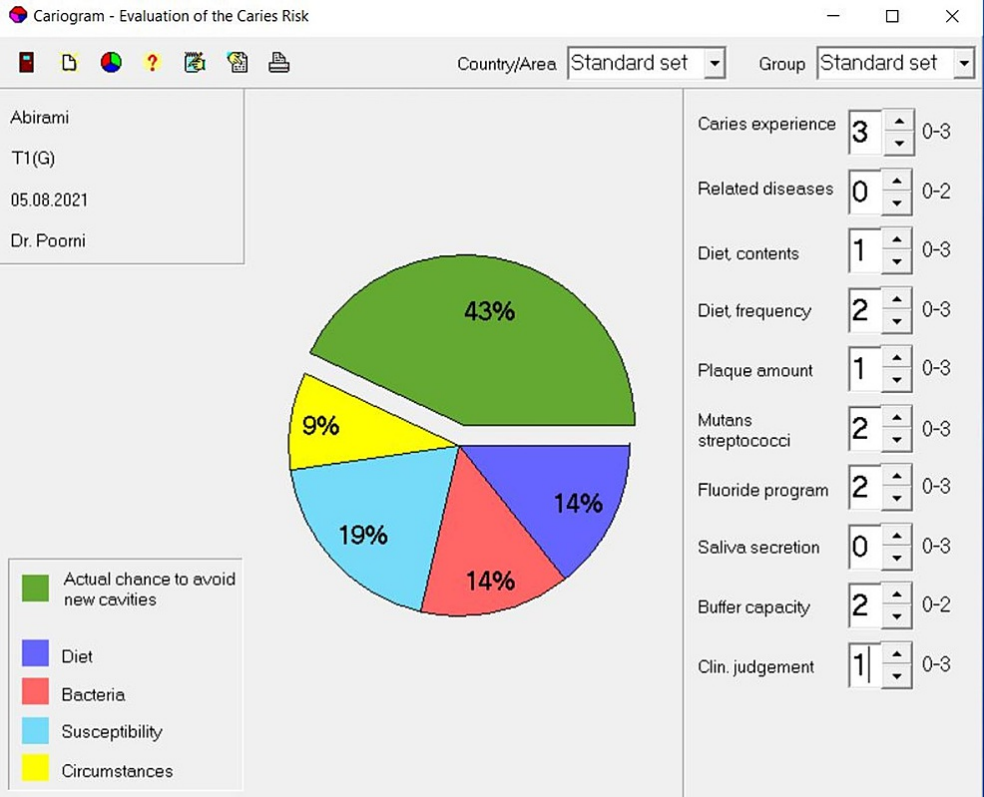
Post-intervention Cariogram Sector (Group 1)

Table 1 shows the comparison of the chance to avoid new cavities among participants between groups and within groups at baseline and three months after the intervention. There was no significant difference in the baseline of the chance to avoid new cavities between the groups (p=0.164). However, there was a significant difference in three months after the intervention of chance to avoid new cavities (p=0.047). There was found to be a statistically significant increase in the chance to avoid new cavities within Group 1 (p=0.001), Group 2 (p=0.001), and Group 3 (p=0.005). The chance to avoid new cavities was found to be high in Group 1 participants with a significant mean percentage increase of 30.93%. Cariogram sectors of baseline and one month after the intervention of one sample in Group 1 is shown in Figures 2, 3.

**Table 1:** Comparison of percentage of chance to avoid new cavities among the participants in intervention groups and control group *Kruskal-Wallis (One-way ANOVA); ^#^ Wilcoxson signed-rank test

Chance to avoid new cavities	Mean ± SD; Median			p-value (between groups)
	Group 1	Group 2	Group 3	
Baseline	16.21 ± 2.665; 16.50	16.79 ± 5.618; 15.50	24.64 ± 10.34; 30.00	0.164*
Three months after the intervention	47.14 ± 6.837; 44.00	41.36 ± 16.05; 45.50	32.50 ± 14.54; 33.00	0.047*
p-value (within groups)	0.001^#^	0.001^#^	0.005^#^	

Table 2 shows a pair-wise comparison of the mean percentage of chance to avoid new cavities. There is a significant difference in mean percentage between Group 1 and Group 3 (p=0.020). There is no significant difference between Group 1 and Group 2 (p=0.654); Group 2 and Group 3 (p=0.059).

**Table 2:** Dunn’s pair-wise comparison of the chance to avoid new cavities between groups in one month after intervention

Pairs	Test statistics	Standard Error	p value
Group 1 vs Group 2	8.714	4.622	0.654
Group 2 vs Group 3	10.786		0.059
Group 3 vs Group 1	2.071		0.020

## Discussion

Caries risk assessment is an essential step to obtain a better understanding of the caries profile of a patient visiting the dental office. Cariogram software has proved to be an effective tool for chairside caries risk evaluation [13]. Cariogram is based on a set of nine criteria that includes the disease indicators, risk factors, and protective factors. The tenth criterion is left to the clinical judgement of the expert dentist. Among the ten factors, the most relevant variable in the prediction of the caries risk is “caries experience” as literature shows a strong relationship between caries experience and caries risk profile [14,15]. Thus, in the present study Cariogram was used to evaluate the change in the caries risk profile following the use of *Streptococcus salivarius* K12 and M18 Lozenges.

*Streptococcus salivarius* is a predominant oral *Streptococcus* species that is not found to be associated with any disease in humans. Literature reveals the effectiveness of this strain on the reduction of dental caries in animal models [16]. This probiotic potential of the *Streptococcus salivarius* may be due to the array of bacteriocins produced along with the exoenzymes dextranase and urease that could limit the caries progress by decreasing the accumulation and acidification of plaque [16].

*Streptococcus salivarius* M18, a strain that has been shown to be capable of inhibiting MS, has relatively broad-spectrum bacteriocin-like inhibitory substance (BLIS) activity against *Streptococcus mutans*. *Streptococcus salivarius* K12 produces two bacteriocins namely, salivaricin A2 and salivaricin B that inhibit the bacterial species that are phylogenetically related. Burton et al. in their study has also demonstrated the beneficial effect of *Streptococcus salivarius* species [17]. These characteristics of K12 and M18 species make them attractive candidates for the prevention of oral diseases. Though there are several studies [16,18] evaluating the effect of these strains on the *Streptococcus* *mutans* count, there are none that has compared the change in the caries risk profiles following the probiotic K12 and M18 use. Thus, in our present study, the effects of these two strains on the Cariogram parameters were evaluated.

The attrition rate was 20% which was not unexpected due to the neglected nature of the population to report after 90 days for assessment. This was taken into consideration during sample size calculation and 14 subjects were enrolled in each group and also multiple imputations was done to avoid outcome reporting bias.

Based on the results of the 90 days treatment with oral probiotics, there was an increase in the chance of avoiding new cavities in the subjects enrolled. The increase in the percentage of the green sector is due to the actual decrease in the other four sectors. The red sector was found to be altered indicating that the interventions mainly altered the *Streptococcus mutans* levels thereby decreasing their caries risk. This could be attributed to the anti-cariogenic properties of the *Streptococcus salivarius* K12 and M18 as discussed earlier. Our earlier study showed that following the use of BLIS K12^TM^ and BLIS M18^TM^ lozenges for 30 days, there was a reduction in the *Streptococcus mutans* count. This may be attributed to the capacity of these strains to colonise the oral tissue [18]. Literature also shows that higher colonisation results in the increased reduction of the cariogenic bacteria in the saliva leading to a reduction in the formation of new carious lesions [19].

The limitation of our study was that the number of participants enrolled for the study and the duration of the study were rather small to arrive at decisive conclusions. Further, larger clinical trials followed up for a longer period of time are essential to draft protocols incorporating the administration of *Streptococcus salivarius* M18 and K12 to reduce the patients’ caries risk.

## Conclusions

Within the limitations of the present clinical study, it can be concluded that the use of BLIS K12^TM ^and BLIS M18^TM^ probiotics for three months resulted in a considerable decrease in the caries risk. Further long-term clinical trials are needed to evaluate the difference in caries risk following the use of BLIS K12^TM^ and BLIS M18^TM^ among different age and risk groups.
